# Efficacy and Safety of Esketamine in Patients Undergoing Laparoscopic Cholecystectomy: A Systematic Review and Meta-Analysis of Randomized Controlled Trials

**DOI:** 10.3390/jcm15134902

**Published:** 2026-06-24

**Authors:** Abdulrahman Hamad Aldousari, Hamad Alkandari, Sulaiman Alruwaished, Yousef M. F. H. Almutairi, Abdulwahab Alkandari, Shekha Alnajdi, Mohammad Alsharah, Salah Termos

**Affiliations:** 1 Faculty of Medicine, University of Jordan, Amman 11942, Jordan; 2 Kuwait Institute for Medical Specialization, Ministry of Health, Kuwait City 13018, Kuwait; 3 Department of General Surgery, The American University of Beirut Medical Center, Beirut 1107 2020, Lebanon; 4 Department of General Surgery, Al-Amiri Hospital, Ministry of Health, Kuwait City 15400, Kuwait

**Keywords:** esketamine, laparoscopic cholecystectomy, systematic review

## Abstract

**Background**: Laparoscopic cholecystectomy is the gold standard for gallbladder disease but is often associated with significant postoperative pain. Opioid analgesia is effective and is the mainstay of treatment. However, opioids are limited by multiple adverse effects such as nausea and respiratory depression. Esketamine is an NMDA receptor antagonist that has emerged as a potential analgesic adjunct. This systematic review and meta-analysis aims to evaluate the efficacy and safety of perioperative esketamine for patients undergoing laparoscopic cholecystectomy. **Methods**: We conducted a systematic review and meta-analysis of randomized controlled trials comparing perioperative esketamine with control regimens in adults undergoing laparoscopic cholecystectomy. Five databases were searched from inception to 25 August 2025. Random-effects meta-analyses were performed for pain, hemodynamic, recovery, and safety outcomes. **Results**: Six randomized controlled trials including 569 patients were eligible. Esketamine was associated with lower pain scores at rest and during movement, although neither were statistically significant. No significant clinical differences were observed in mean arterial pressure or heart rate changes during surgery or after surgery. However, esketamine significantly shortened wake-up time (MD = −3.55 min, 95% CI [−6.09 to −1.02]), improved postoperative sleep quality (MD = −5.78, 95% CI [−6.80 to −4.76]), and reduced PONV (RR = 0.47, 95% CI [0.24 to 0.92]) and respiratory depression (RR = 0.18, 95% CI [0.03 to 0.98]). **Conclusions**: Esketamine improved selected recovery and safety outcomes but did not significantly reduce hemodynamic parameters, postoperative pain or rescue analgesia. Larger high-quality trials are needed to confirm its role in laparoscopic cholecystectomy.

## 1. Introduction

Laparoscopic cholecystectomy is considered as the gold standard surgical procedure for gallbladder disease owing to its relatively short operative duration, improved visualization, minimal tissue trauma, reduced intraoperative bleeding, accelerated postoperative recovery, and low infection risk [1,2]. However, a lot of people still experience severe pain just after laparoscopic cholecystectomy, even with current improvements. This pain is an immediate nociceptive reaction that could produce a lot of physical discomfort, unstable blood flow and metabolism, a longer recovery time, and a delayed discharge from the hospital. The pain that comes after laparoscopic cholecystectomy is complicated and mostly made up of incisional pain, visceral discomfort, and hyperalgesia brought on by opioids [3,4,5].

Opioids are still the most significant part of pain relief before and after surgery since they work very well to ease pain, lower the body’s stress response to operation, and protect organs, especially when the pain is moderate to severe [6]. However, opioid-based analgesic methods can cause a lot of side effects, like nausea, vomiting, constipation, central sensitization, opioid-induced hyperalgesia, and possible immunosuppressive effects, which could worsen recovery after surgery [7,8,9,10].

Weak opioid or opioid-sparing anesthesia and analgesia have been popular in the last several years as good ways to control pain before, during, and after surgery. Esketamine is a novel anesthetic medicine that functions as both a sedative and a painkiller. Esketamine, which is the S-enantiomer of ketamine, binds to N-methyl-D-aspartate (NMDA) receptors three to four times more strongly than racemic ketamine. It also has less of a deleterious effect on the heart and lungs [11,12]. Esketamine can rise blood pressure, open up the airways, lessen the resistance in the airways after surgery, and make breathing easier by inhibiting NMDA receptors and turning on the sympathetic nervous system [13]. Esketamine has been shown to reduce postoperative pain intensity and decrease the frequency of sleep disturbances when administered in low continuous doses [14].

Esketamine’s analgesic and opioid-sparing properties are increasingly acknowledged; nevertheless, evidence supporting its effectiveness remains inadequate. Additionally, the impact of esketamine on pain management, postoperative recovery, and hemodynamic responses in laparoscopic cholecystectomy patients is not well elucidated, and there is a lack of established clinical guidelines for its use in this surgical context. The aim of this systematic review and meta-analysis is to evaluate the efficacy and safety of esketamine in a perioperative analgesic context for patients undergoing laparoscopic cholecystectomy.

## 2. Methods

### 2.1. Review Protocol Registration

The study was conducted in accordance with the methodological guidance outlined in the Cochrane Handbook for Systematic Reviews of Interventions and reported following the Preferred Reporting Items for Systematic Reviews and Meta-Analyses (PRISMA) standards. This review was registered in PROSPERO under registration number CRD420261379009. The PRISMA 2020 checklist is provided in the Supplementary Materials (Table S1). Ethical approval was not required, as the analysis relied exclusively on data from previously published studies.

### 2.2. Search Strategy

A comprehensive literature search was performed using the electronic databases PubMed, Scopus, Web of Science, EMBASE, and Cochrane Central Register of Controlled Trials (CENTRAL) from database inception to 25 August 2025. Our search strategy included (“Laparoscopy” or “Laparoscopic” or “Peritoneoscopy”) AND (“Cholecystectomy” or “gallbladder removal” or “gallbladder surgery”) AND (“Esketamine” or “Kataved” or “Spravato” or “(S)-2-(o-chlorophenyl)-2-(methylamino)cyclohexanone” or “L-Ketamine”). Additionally, the list of retrieved articles was manually searched to ensure that no articles were missed. The full search strategies for each database are presented in Table S2. No restrictions were applied on the basis of country of origin or publication status. Authors of studies with incomplete or unclear data were contacted for clarification when necessary.

### 2.3. Eligibility Criteria

The eligibility criteria were defined according to the PICO framework. Studies were considered eligible if they included adult patients undergoing laparoscopic cholecystectomy and evaluated the perioperative use of esketamine as part of anesthesia or analgesia. Eligible comparators included placebo, no esketamine, or conventional opioid-based or opioid-sparing anesthetic regimens. Outcomes of interest comprised postoperative pain scores; hemodynamic parameters such as mean arterial pressure (MAP) and heart rate (HR); and recovery-related measures, including wake-up time, recovery time, duration of surgery and anesthesia, and the need for rescue analgesia. Additionally, studies reporting adverse events such as postoperative nausea and vomiting (PONV), hallucinations, pruritus, dizziness, nightmares, and respiratory depression were considered. Only randomized controlled trials (RCTs) were included.

Studies were excluded if they evaluated esketamine in combination with other drugs, were observational or non-randomized in design, involved animal subjects, were available only as abstracts without full-text access, or were review articles or case reports. Studies published in languages other than English were also excluded.

### 2.4. Study Selection and Data Extraction

All retrieved records were imported into EndNote software, where duplicate citations were identified and removed. Study selection was conducted in two stages. First, titles and abstracts were screened for relevance. Second, full texts of potentially eligible articles were independently assessed for final inclusion. Two reviewers performed the screening process independently, and any discrepancies were resolved through discussion and consensus.

Data extraction was independently performed by two reviewers using a standardized data extraction form. Extracted study characteristics included author name, publication year, country, sample size, study design, and intervention details. Baseline patient characteristics included age, sex distribution, body mass index, American Society of Anesthesiologists (ASA) classification, duration of surgery and anesthesia, pain assessment tool used, and follow-up duration. Detailed anesthesia and surgical protocols were also extracted to assess clinical comparability between studies, including pre-medications, anesthesia protocols, surgical protocols, and post-medications.

Our primary endpoints were perioperative hemodynamic changes in mean arterial pressure (MAP, mmHg) and heart rate (HR, bpm), analyzed as change scores calculated from baseline to during surgery values and from baseline to after surgery values. Secondary endpoints included emergence and recovery parameters, including duration of anesthesia (minutes), duration of surgery (minutes), and wake-up time (minutes). Additional secondary endpoints included postoperative pain intensity measured using validated 10-point pain scales (Visual Analog Scale [VAS] or Numeric Rating Scale [NRS]) at resting and at movement (cough) and incidence of rescue analgesic requirement (%). Safety outcomes included the incidence of PONV (%), hallucinations (%), pruritus (%), dizziness (%), nightmares (%), and respiratory depression (%).

Dichotomous outcomes were extracted in event/total format, and continuous outcomes were extracted as means with standard deviations. In cases where studies reported medians with interquartile ranges or ranges, the conversion formulas by Wan et al. were used in an attempt to estimate means and standard deviations. For trials reporting outcomes at multiple time points, data at every clinically relevant time point were extracted based on a predefined outcome framework used in this review.

### 2.5. Risk of Bias Assessment

The methodological quality of the included RCTs was assessed using the Cochrane Risk of Bias tool version 2 (RoB 2) [15]. The following domains were evaluated: bias arising from the randomization process, bias due to deviations from intended interventions, bias due to missing outcome data, bias in measurement of outcomes, and bias in selection of reported results. Each domain was rated as low risk, some concerns, or high risk of bias. Two reviewers independently conducted the risk of bias assessment, and disagreements were resolved by consensus. Owing to the limited number of included studies for most outcomes, formal assessment of publication bias using funnel plots or Egger’s regression test was not considered statistically reliable and therefore was not performed, in accordance with Cochrane recommendations.

### 2.6. Statistical Analysis

Statistical analyses were performed using RevMan, 5.4 version and RStudio, 4.6 version. Continuous outcomes were analyzed using mean differences (MDs), while standardized mean differences (SMDs) were applied when different measurement scales were used across studies. Dichotomous outcomes were evaluated using risk ratios (RRs). All pooled effect estimates were reported with corresponding 95% confidence intervals (CIs), and a *p*-value ≤ 0.05 was considered statistically significant.

Given the anticipated clinical heterogeneity related to variations in esketamine dosing, timing of administration, and anesthesia protocols, a random-effects model was applied for all analyses. Statistical heterogeneity was assessed using the Chi-square test and quantified using the I^2^ statistic, with I^2^ values of <25%, 25–50%, and >50% representing low, moderate, and substantial heterogeneity, respectively. Substantial heterogeneity was additionally considered when the Chi-square *p*-value was <0.10. For outcomes with zero-event cells, a continuity correction of 0.5 was applied. In studies with multiple intervention arms, each esketamine arm was treated as an independent comparison and analyzed against the same control group. Sensitivity analyses were not performed because of the limited number of included studies and the small sample sizes available for most pooled outcomes.

## 3. Results

### 3.1. Systematic Literature Search

**Figure 1:**
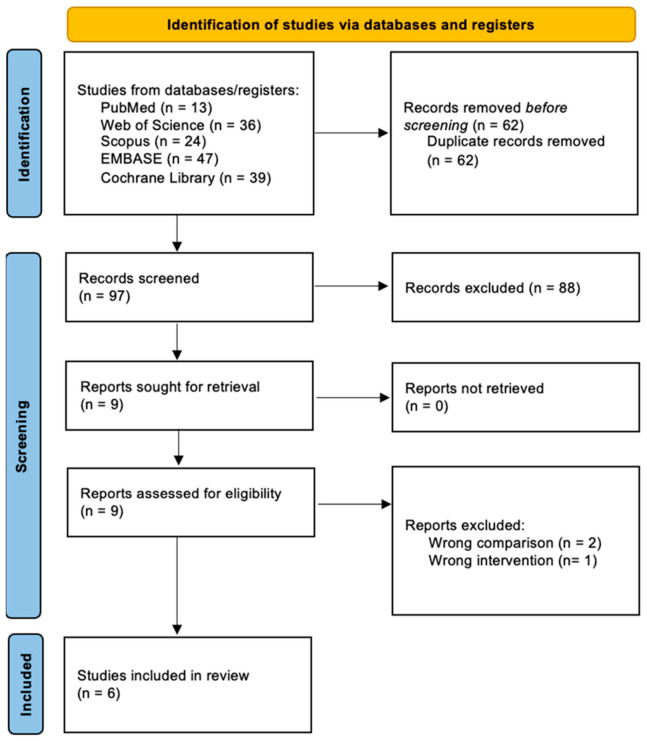
The Preferred Reporting Items for Systematic reviews and Meta-Analyses (PRISMA) flow chart of the screening process.

The initial search identified 159 records across all databases, including manual searching of reference lists and academic platforms. Titles and abstracts were screened to remove ineligible studies, during which duplicate records and non-relevant articles were excluded. Following this preliminary screening, nine studies were selected for full-text assessment. During full-text assessment, three studies were excluded for not meeting the predefined eligibility criteria, as detailed in Table S3. Ultimately, six randomized controlled trials [16,17,18,19,20,21] met the eligibility criteria and were included in the quantitative synthesis, as shown in Figure 1.

### 3.2. Summary and Baseline Characteristics of the Included RCTs

A total of six RCTs were included in the final analysis, encompassing a combined population of 569 patients. Across studies, participants were generally middle-aged adults, with mean ages ranging from approximately 47 to 55 years and a balanced male-to-female distribution. Baseline characteristics were comparable between treatment groups in each study. A summary of individual study characteristics and baseline demographics is provided in Table 1 and Table 2. The detailed surgical and anesthetic characteristics are provided in Table S4.

### 3.3. Pain Score Assessment

**Figure 2:**
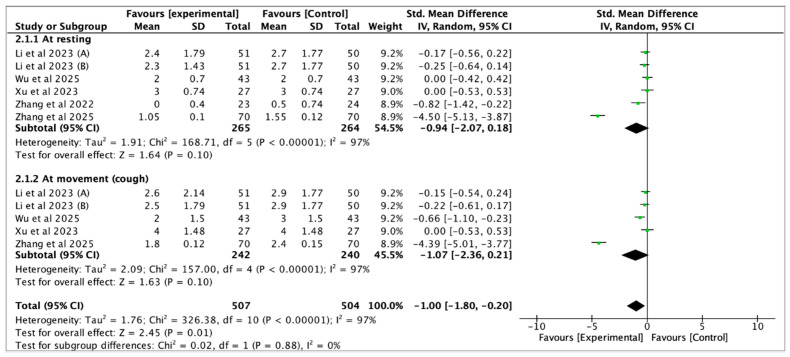
Forest plots of the meta-analysis showing the SMD in postoperative pain (10-point) at resting and at movement (cough).

A total of 327 patients were included in this outcome. There was no statistically significant difference between the two groups in the pain score at resting (*n* = 5, SMD = −0.94, 95% CI [−2.07 to 0.18], *p* = 0.10) and at movement (SMD = −1.07, 95% CI [−2.36 to 0.21], *p* = 0.10), with a substantial heterogeneity (I^2^ = 97%), Figure 2.

### 3.4. Mean Arterial Pressure (MAP)

**Figure 3:**
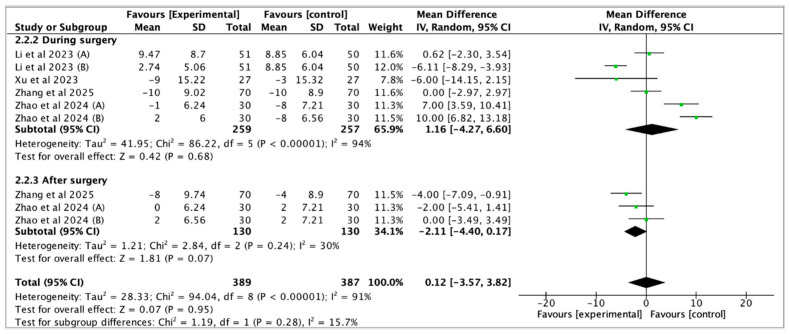
Forest plots of the meta-analysis showing the MD in MAP (mmHg) during surgery and after surgery.

There was no statistically significant difference between the two groups in terms of MAP during surgery (*n* = 4 RCTs with 436 patients, MD = 1.16, 95% CI [−4.27 to 6.60], *p* = 0.1) and after surgery (*n* = 2 RCTs with 230 patients, MD = −2.11, 95% CI [−4.40 to 0.17], *p* = 0.1). Although there is moderate heterogeneity between the studies included in after surgery subgroup (I^2^ = 30), the heterogeneity across studies included in during surgery subgroup were substantial (I^2^ = 94%), Figure 3.

### 3.5. Heart Rate

**Figure 4:**
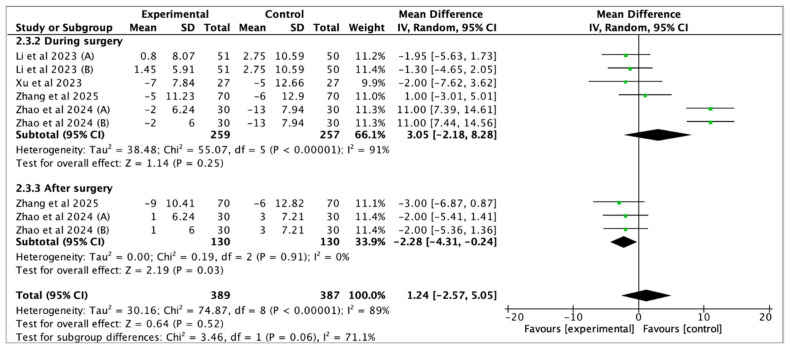
Forest plots of the meta-analysis showing the MD in HR (bpm) during surgery and after surgery.

There was no statistically significant difference between the two groups in terms of HR during surgery (*n* = 4 RCTs with 436 patients, MD = 3.05, 95% CI [−2.18 to 8.28], *p* = 0.25) with substantial heterogeneity (I^2^ = 91%). However, the HR after surgery was statistically significantly lower in esketamine group (*n* = 2 RCTs with 230 patients, MD = −2.28, 95% CI [−4.31 to −0.24], *p* = 0.03) with low heterogeneity (I^2^ = 0%), Figure 4.

### 3.6. Wake-Up Time

A total of 144 patients were included in this outcome. Time to awakening was significantly shorter in the esketamine group (*n* = 2, MD = −3.55, 95% CI [−6.09 to −1.02]; *p* = 0.006). The heterogeneity was substantial (I^2^ = 85%), as shown in Figure S1.

### 3.7. Recovery Time

A total of 187 patients were included in this outcome. There was no significant difference between the two groups in terms of recovery time (*n* = 2, MD = 1.74, 95% CI [−3.79 to 7.28]; *p* = 0.54). The heterogeneity was substantial (I^2^ = 86%), as shown in Figure S2.

### 3.8. Duration of Surgery

**Figure 5:**
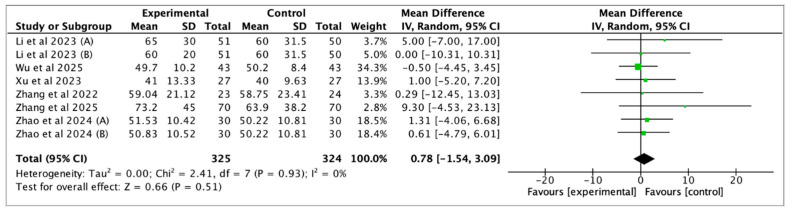
Forest plots of the meta-analysis showing the MD in duration of surgery.

A total of 569 patients were included in this outcome. There was no significant difference between the two groups in terms of duration of surgery (*n* = 6, MD = 0.78, 95% CI [−1.54 to 3.09]; *p* = 0.51). The heterogeneity was low (I^2^ = 0%), as shown in Figure 5.

### 3.9. Duration of Anesthesia

**Figure 6:**
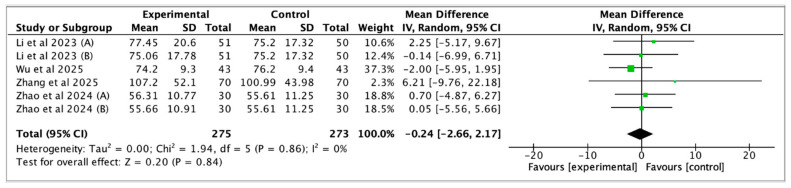
Forest plots of the meta-analysis showing the MD in duration of anesthesia.

A total of 468 patients were included in this outcome. There was no significant difference between the two groups in terms of duration of anesthesia (*n* = 4, MD = −0.24, 95% CI [−2.66 to 2.17]; *p* = 0.84). The heterogeneity was low (I^2^ = 0%), Figure 6.

### 3.10. Sleep Quality

A total of 238 patients were included in this outcome. Sleep quality was better in the esketamine group (*n* = 2, MD = −5.78, 95% CI [−6.8 to −4.76]; *p* < 0.00001). The heterogeneity was low (I^2^ = 0%), as shown in Figure S3.

### 3.11. Postoperative Nausea and Vomiting (PONV)

A total of 331 patients were included in this outcome. The esketamine group had significantly lower PONV incidence compared to the control group (*n* = 4, RR = 0.47, 95% CI [0.24 to 0.92]). The heterogeneity was low (I^2^ = 0%), as shown in Figure S4.

### 3.12. Hallucination

A total of 254 patients were included in this outcome. The pooled analysis demonstrated no significant difference between the two groups (*n* = 3, RR = 1.00, 95% CI [0.11 to 9.46]). The heterogeneity was low (I^2^ = 0%), as shown in Figure S5.

### 3.13. Itching

A total of 194 patients were included in this outcome. There was no significant difference between the two groups in terms of itching incidence (*n* = 3, RR = 0.6, 95% CI [0.08 to 4.46]). The heterogeneity was low (I^2^ = 0%), as shown in Figure S6.

### 3.14. Nightmare

A total of 296 patients were included in this outcome. There was no significant difference between the two groups in terms of nightmare (*n* = 3, RR = 2.77, 95% CI [0.66 to 11.61]). The heterogeneity was low (I^2^ = 0%), as shown in Figure S7.

### 3.15. Respiratory Depression

A total of 144 patients were included in this outcome. The esketamine group had a significantly lower respiratory depression rate compared to the control group (*n* = 2, RR = 0.18, 95% CI [0.03 to 0.98]). The heterogeneity was low (I^2^ = 0%), as shown in Figure S8.

### 3.16. Dizziness

A total of 346 patients were included in this outcome. There was no significant difference between the two groups in terms of dizziness (*n* = 3, RR = 1.81, 95% CI [0.76 to 4.31]). The heterogeneity was low (I^2^ = 0%), as shown in Figure S9.

### 3.17. Requiring Rescue Analgesia

A total of 280 patients were included in this outcome. There was no significant difference between the two groups in terms of requiring rescue analgesia (*n* = 3, RR = 0.72, 95% CI [0.42 to 1.26]). The heterogeneity was moderate (I^2^ = 45%), as shown in Figure S10.

### 3.18. Risk of Bias Assessment

**Figure 7:**
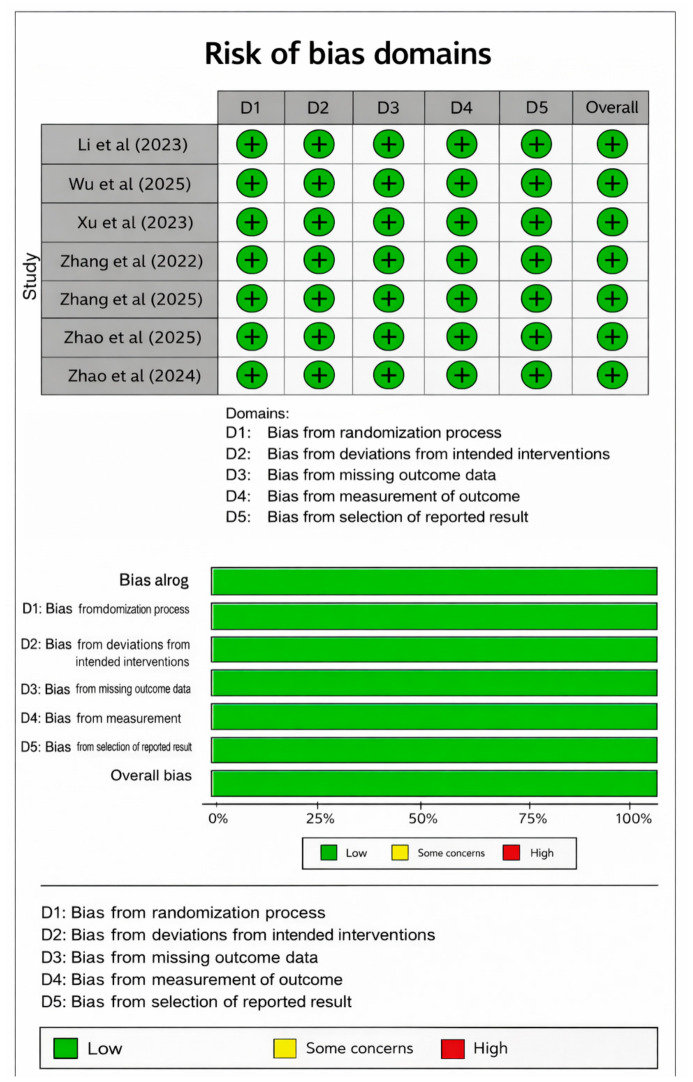
Risk of bias summary and graph for the included randomized trials. The upper panel displays the risk of bias assessment for each individual study, categorized as low (green), some concerns (yellow), or high (red). The lower panel summarizes the risk of bias across all included studies for each bias domain.

Six trials demonstrated a low risk of bias across all assessed domains, indicating generally favorable methodological quality. Nevertheless, the relatively small sample sizes and variability in perioperative protocols across studies should still be considered when interpreting pooled effect estimates. A graphical summary of domain-level assessments and study-level ratings is presented in Figure 7.

## 4. Discussion

### 4.1. Summary of Findings

This systematic review and meta-analysis found that perioperative esketamine was associated with selected recovery and safety benefits, including shorter wake-up time, improved postoperative sleep quality, and reduced PONV and respiratory depression. However, esketamine did not significantly reduce postoperative pain scores or rescue analgesia requirements. In terms of intraoperative hemodynamic stability, there was no clinically significant difference between the two groups. No statistically significant differences were identified between groups in other neuropsychiatric adverse events, including hallucinations, nightmares, and dizziness.

### 4.2. Analgesic Efficacy

Our research demonstrated that esketamine used perioperatively improved postoperative pain scores and decreased the amount of rescue medication used after laparoscopic cholecystectomy; however, these numbers are not statistically significant and do not provide conclusive evidence for using ketamine to provide multimodal analgesia. Although a trend favoring esketamine was observed, the currently available evidence remains insufficient to confirm a clinically meaningful analgesic benefit in this surgical setting. The findings in our report differ from those found in a meta-analysis by Akram et al. [22], which showed statistically significant decreases in both postoperative pain and the need for rescue medication when using ketamine and esketamine perioperatively during laparoscopic cholecystectomy. There are multiple reasons for the discrepancy in the findings. First, the meta-analysis by Akram et al. combined the results of studies utilizing both ketamine and esketamine into one model, and our analysis specifically evaluated patients receiving esketamine only for their interventions. Second, the studies included in our analysis differ substantially regarding dosing methods, timing of medication administration, type of anesthetics utilized, and analgesia postoperative regimens. Third, our analysis included only randomized controlled trials and focused on laparoscopic cholecystectomy as the only surgical procedure studied reducing heterogeneity but limiting the number of participants. Furthermore, several studies included utilized multimodal analgesic techniques with local anesthetic infiltration or regional blocks and as a result may have reduced the overall effect of esketamine as an independent analgesic. The findings of Mogianos et al. are consistent with this uncertainty, as their trial revealed no significant differences in rescue opioid requirements and pain scores between an esketamine-containing multimodal regimen and standard care [23].

### 4.3. Hemodynamic Stability

Our study found that esketamine did not result in clinically significant hemodynamic instability, as MAP and HR were broadly comparable with control during the surgery. This aligns with a systematic review and meta-analysis conducted by Wang et al., which reported no significant change in postoperative MAP or HR with esketamine compared with standard care [24]. Although the pooled analysis showed a statistically significant reduction in postoperative heart rate in the esketamine group, the magnitude of this difference was small and is unlikely to be clinically meaningful. Therefore, despite statistical significance, this finding should be interpreted cautiously and does not suggest clinically relevant hemodynamic instability. Physiologically, esketamine has a sympathomimetic action that tends to maintain or slightly elevate blood pressure. A recent randomized controlled trial in critically ill patients undergoing emergent intubation found that esketamine induction resulted in higher MAP than a midazolam/sufentanil regimen, while HR remained unchanged [25]. In many clinical anesthesia contexts, this slight elevation of MAP is considered beneficial. Zhang et al. highlighted that esketamine can counteract circulatory depression and hypotensive episodes commonly induced by other induction agents such as propofol or midazolam [26]. By preserving cardiac output and forward flow, esketamine may provide better hemodynamic stability in patients who are hemodynamically compromised or elderly. The absence of a significant difference in MAP and HR after surgery in our analysis suggests that these effects are transient and do not translate into sustained postoperative hypertension or tachycardia in otherwise stable adults undergoing laparoscopic cholecystectomy.

### 4.4. Recovery

Our analysis of recovery showed a distinct dichotomy: time to awakening (wake-up time) was significantly shorter in the esketamine group, whereas overall recovery time showed no statistically significant difference. The faster awakening observed with esketamine may be explained by its rapid distribution half-life and high clearance rate (18.1 mL/min/kg), which limit drug accumulation during short procedures [27]. Furthermore, because esketamine provides potent analgesia, it may reduce the required doses of other hypnotics such as propofol and longer-acting opioids [28,29]. Conversely, evidence from other surgical populations has shown inconsistent effects of esketamine on postoperative recovery profiles. Some research has reported a quicker recovery time and better quality of recovery with the use of esketamine [29]; however, other studies have documented a longer recovery period attributed to residual sedation/disassociation effects when using higher doses [30,31]. Zhu et al. found that recovery time was significantly longer in patients undergoing modified radical mastectomy who received intraoperative esketamine [30]. This apparent discrepancy may be explained by the dose-dependent dissociative effects of ketamine derivatives. At higher subanesthetic doses, or during prolonged procedures involving extended esketamine infusions, residual dissociation or sedation may delay full cognitive recovery [31]. Thus, while patients may open their eyes sooner because of a reduced hypnotic burden, the overall time required to resolve dissociative effects and satisfy post-anesthesia care unit discharge criteria may remain unchanged.

### 4.5. Safety and Adverse Events

Our analysis showed that esketamine significantly reduced the incidence of postoperative nausea and vomiting (PONV). This finding may be clinically relevant because reduction of PONV can contribute to improved postoperative recovery, earlier mobilization, and greater patient satisfaction following ambulatory laparoscopic surgery. Opioids are well-known triggers of PONV because they activate mu-opioid receptors in the chemoreceptor trigger zone and vestibular pathways [32]. Furthermore, opioids delay gastric emptying and inhibit acetylcholine release in the gastrointestinal tract, which can contribute to nausea and postoperative ileus [33]. By providing non-opioid analgesia through NMDA receptor blockade, esketamine may allow clinicians to reduce intraoperative and postoperative doses of remifentanil and sufentanil [29]. Our results regarding PONV are relatively consistent with other literature documenting perioperative esketamine. Randomized studies and recent meta-analyses have indicated that patients receiving perioperative esketamine had lower rates of PONV, especially when there was a concurrent reduction in the use of opioids. [34,35]. A large-scale meta-analysis of esketamine in surgical patients confirmed a significant reduction in nausea and vomiting and a shorter time to first flatus [34]. Similarly, a trial in gynecological laparoscopy demonstrated that a single dose of esketamine (0.25 mg/kg) at incision closure significantly reduced both the incidence and severity of PONV [35]. This supports the theory that esketamine’s ability to reduce the use of opioids may indirectly lead to improved gastrointestinal recovery and reduced emetic exposure.

Regarding respiratory depression, our pooled analysis indicated a significant reduction associated with esketamine. Unlike opioids and hypnotics such as propofol, which are potent respiratory depressants that shift the CO_2_ response curve to the right and decrease tidal volume, esketamine generally preserves or may even stimulate spontaneous respiration [36]. In addition, esketamine better preserves upper airway reflexes, including swallowing and coughing, than many other intravenous anesthetics, thereby reducing aspiration risk [37]. These properties make esketamine a valuable option in patients who are vulnerable to respiratory compromise.

Differences between the two groups in the incidence of hallucinations or nightmares were not significant in our pooled analysis. However, a systematic review and meta-analysis including ketamine and esketamine reported a higher incidence of hallucinations in the intervention group [22]. These findings may be influenced by dose and context: high doses or continuous infusions are more likely to cause vivid dreams or dissociative symptoms, whereas single low-dose boluses are often well tolerated. Serious postoperative nightmares or hallucinations were rare across the included studies and were generally transient. The relatively low frequency of these adverse effects in our review may reflect the fact that most included trials used esketamine at subanesthetic doses within balanced anesthetic techniques based on propofol or midazolam [38]. Furthermore, interpretation of neuropsychiatric safety outcomes remains limited because several adverse events, including sedation-related outcomes, were inconsistently reported across the included trials.

Overall, the current literature suggests that perioperative esketamine may provide greater benefit in recovery quality and opioid-related adverse event reduction than in direct postoperative pain reduction alone. This distinction is important because improved recovery outcomes, including lower PONV incidence, preserved respiratory function, and improved sleep quality, may still carry substantial clinical value even in the absence of statistically significant reductions in pain scores.

### 4.6. Clinical Implications

The clinical implications of these findings are important for anesthesiologists and perioperative care teams, although they should be interpreted cautiously. In patients undergoing laparoscopic cholecystectomy, perioperative esketamine was not associated with a statistically significant reduction in postoperative pain scores or rescue analgesia requirements. Thus, statistically significant findings should not automatically be interpreted as clinically transformative, particularly when pooled effect sizes remain modest. However, there were other recovery and safety advantages of using this drug, including faster awakening times, improvement of patients’ sleep quality, and reduced risks of PONV and respiratory depression.

Based on currently available results, esketamine can still be used in combination with other drugs within a multimodal anesthesiology protocol to spare opioids and achieve additional effects. Though the risk of hemodynamic instability and delayed recovery was not observed in a pooled analysis, the drug remains safe for this particular type of surgery. These findings may support the integration of esketamine into opioid-sparing enhanced recovery after surgery pathways in selected patients. Nevertheless, the small sample size and heterogeneity of dosing regimes do not allow one to recommend the routine application of the drug. Clinicians should individualize esketamine administration, monitor for dose-related adverse effects such as dissociation or sedation, and await further high-quality trials to better define its optimal role in laparoscopic cholecystectomy.

### 4.7. Strengths and Limitations

Our systematic review and meta-analysis had several strengths. We included only RCTs, and we performed a rigorous risk of bias assessment. The majority of the included trials were high-quality randomized, double-blinded studies with low risk of bias in most domains. We also reported a comprehensive set of outcomes; beyond pain, we examined hemodynamic and recovery parameters, allowing a balanced interpretation of benefits and risks. There are a few weaknesses that need to be acknowledged. First, although the included trials were generally of favorable methodological quality, small-study effects and protocol variability may still influence the robustness of pooled estimates. Second, the trials used different doses of esketamine varying from 0.2 to 0.75 mg/kg, administration methods included single bolus and infusion, and certain trials combined esketamine with other medications such as nalbuphine or dexmedetomidine, while others did not. Clinical heterogeneity existed in the included studies because of differences in the dosage regimens for esketamine, timing of administration (whether before or after the surgery), methods of anesthesia, types and dosage of analgesia provided, and ways of measuring outcomes. These differences potentially contributed to a high degree of statistical heterogeneity that was observed in multiple pooled analyses. Random-effects models were used to help mitigate the effects of expected clinical heterogeneity; however, there remains the possibility that the residual interstudy variability may influence both the precision and interpretability of pooled effect estimates. Third, the relatively short postoperative follow-up durations limited the assessment of long-term outcomes such as chronic pain and persistent opioid use. Additionally, there were additional outcome measures that were potentially significant in evaluating perioperative safety, including sedation-related parameter data and neuropsychiatric-related adverse events, that were not consistently reported throughout the studies and that would limit the totality of pooled safety data analyses emitted from this study. Fourth, although we did not detect obvious publication bias, the number of studies is small, so we cannot rule it out. Finally, the limited number of studies available for several outcomes reduced the feasibility of conducting subgroup or sensitivity analyses.

### 4.8. Recommendations for Practice and Research

Based on the current evidence, we recommend considering incorporating low-dose esketamine as a part of a multimodal analgesia regimen for laparoscopic cholecystectomy. Typical regimens include a single bolus (e.g., 0.3–0.5 mg/kg) at induction or a low-dose infusion intraoperatively. Practitioners should keep an eye on hemodynamics and the level of sedation when administering esketamine; however, current evidence suggests that it does not produce significant hypotension or delayed awakening. Esketamine is a viable option for people who wish to stay away from opioids, and it might make patients feel better and more satisfied. These positive results should form the basis for future trials. Multicenter RCTs are needed to find the right dosing regimens. Studies should use standardized outcome measures (e.g., 0–10 VAS at set intervals and total opioid consumption in morphine equivalents) to allow clearer comparisons and meta-analysis. Investigators should also examine broader outcomes including cognitive recovery and inflammatory markers. Given the observed reduction in PONV, future larger trials should confirm whether this effect is mediated by reduced opioid exposure or by other perioperative mechanisms. Long-term follow-up could assess whether intraoperative esketamine has effects on chronic pain or opioid dependence. Finally, cost-effectiveness analyses could determine if the improved pain control translates to shorter hospital stays or reduced resource use.

## 5. Conclusions

Perioperative esketamine may provide selected recovery and safety benefits in patients undergoing laparoscopic cholecystectomy, including shorter wake-up time, improved sleep quality, and reduced PONV and respiratory depression. However, it did not significantly reduce hemodynamic parameters, postoperative pain scores or rescue analgesia requirements. Given the limited number of trials and substantial heterogeneity in some outcomes, further well-designed RCTs are needed to confirm its clinical benefit and optimal dosing. Overall, esketamine appears to be a suitable component of opioid-sparing anesthesia strategies. Further research is warranted to optimize dosing strategies and better define its long-term benefits in perioperative care.

## Figures and Tables

**Table 1:** Summary information of the included randomized controlled trials.

Study ID	Trial Registration ID	Study Design	Country	Sample Size (*n*)	Trial Duration	Esketamine Regimen	Control	Surgical Procedure	Anesthesia	Local Anesthesia	Follow-Up Duration
Li et al. (2023) [16]	ChiCTR2100054508	RCT	China	153	December 2021–August 2022	Group A: 0.2 mg/kg/h 1 min before intubation Group B: 0.3 mg/kg/h 1 min before intubation	Placebo (normal saline)	Laparoscopic cholecystectomy	TIVA	NR	48 h
Wu et al. (2025) [17]	ChiCTR2400083826	RCT	China	86	12 May 2024–31 August 2024	0.5 mg/kg/h was administered during the operation and stopped 30 min before the end of the surgery	Placebo (normal saline)	Laparoscopic cholecystectomy	TIVA	Local infiltration (20 mL of 0.375% ropivacaine)	72 h
Xu et al. (2023) [18]	ChiCTR2200066663	RCT	China	54	NR	Different doses were given intravenously; the starting dose of esketamine was set at 0.3 mg/kg	Placebo (normal saline)	Laparoscopic cholecystectomy	Balanced anesthesia	Local infiltration (0.5% ropivacaine)	NR
Zhang et al. (2025) [20]	ChiCTR2400079362	RCT	China	140	15 January 2024–15 April 2024	0.3 mg/kg was given	Placebo (normal saline)	Laparoscopic cholecystectomy	Balanced anesthesia	TAPB (20 mL of 0.375% ropivacaine)	72 h
Zhang et al. (2022) [19]	ChiCTR2000040077	RCT	China	47	December 2020–March 2021	0.1 mL/kg was administered during anesthetic induction	Placebo (normal saline)	Laparoscopic cholecystectomy	TIVA	NR	NR
Zhao et al. (2024) [21]	ChiCTR2300067596	RCT	China	90	February 2022–January 2023	Group A: 0.5 mg/kg was administered initially before skin incision, followed by 2 μg/kg·min Group B: 0.5 mg/kg was administered initially before skin incision, followed by 4 μg/kg·min	Placebo (normal saline)	Laparoscopic cholecystectomy	TIVA	NR	24 h

**Abbreviations:** RCT = randomized controlled trial, TIVA = total intravenous anesthesia, NR = not reported.

**Table 2:** Baseline characteristics of the included participants and studies.

Study ID	Group	N	Age (Years)	Sex (*n*) [Male/Female]	ASA (*n*) [I/II/III]	BMI (kg/m ^2^)	DM, *n* (%)	HTN, *n* (%)	Pain Tool	Sleep Quality Tool
Li et al. (2023) [16]	Esketamine (A)	51	49 (39–54) *	[19/32]	[32/19/0]	24.13 ± 2.36	5 (9.8)	9 (17.6)	NRS	AIS
	Esketamine (B)	51	47(39–55) *	[20/31]	[28/23/0]	24.35 ± 2.48	6 (11.8)	10 (19.6)		
	Placebo/Control	50	53 (37.25–57.25) *	[16/34]	[28/22/0]	24.65 ± 2.68	3 (6)	8 (16)		
Wu et al. (2025) [17]	Esketamine	43	47.9 ± 9.4	[23/20]	[20/23/0]	24.5 ± 3.5	1 (2.3)	3 (7)	VAS	AIS
	Placebo/Control	43	49.7 ± 15.1	[21/22]	[17/23/3]	25.6 ± 2.8	1 (2.3)	7 (16.3)		
Xu et al. (2023) [18]	Esketamine	27	56 (41–67) *	[8/19]	[11/16/0]	23.74 (23.15–24.46) *	NR	NR	VAS	NR
	Placebo/Control	27	55 (51–61) *	[9/18]	[13/14/0]	22.86 (22.1–24.57) *				
Zhang et al. (2025) [20]	Esketamine	70	46.4 ± 13.7	[36/34]	[14/56/0]	24.96 ± 5.97	4 (5.7)	6 (8.6)	VAS	NR
	Placebo/Control	70	47.5 ± 15.8	[54/16]	[22/45/3]	24.05 ± 4.79	5 (7.1)	5 (7.1)		
Zhang et al. (2022) [19]	Esketamine	23	51.57 ± 11.96	[13/10]	NR	24.31 ± 3.70	NR	NR	NRS	NR
	Placebo/Control	24	51.04 ± 8.35	[11/13]	NR	25.01 ± 4.59				
Zhao et al. (2024) [21]	Esketamine (A)	30	40.3 ± 10.3	[16/14]	[15/15/0]	22.4 ± 1.6	NR	NR	NRS	NR
	Esketamine (B)	30	39.5 ± 10.6	[14/16]	[14/16/0]	22.8 ± 1.5				
	Placebo/Control	30	39.3 ± 11.2	[15/15]	[16/14/0]	22.5 ± 1.7				

Abbreviations: ASA = American Society of Anesthesiologists, BMI = body mass index, NRS = Numeric Rating Scale, VAS = Visual Analog Scale, NR = not reported; * indicates median (interquartile range), DM = diabetes mellitus, HTN = hypertension.

## Data Availability

The data supporting the findings of this study are available within the article and Supplementary Materials. Further inquiries can be directed to the corresponding author.

## Supplementary Materials

The following supporting information can be downloaded at: https://www.mdpi.com/article/10.3390/jcm15134902/s1, Table S1. PRISMA 2020 checklist Table S2. Detailed search strategy for each database during the systematic search phase. Table S3. List of excluded studies during the full-text screening step. Table S4. Detailed summary of anesthesia and surgical protocols of the included trials. Figure S1. Forest plots of the meta-analysis showing the MD in wake-up time. Figure S2. Forest plots of the meta-analysis showing the MD in recovery time. Figure S3. Forest plots of the meta-analysis showing the MD in sleep quality score. Figure S4. Forest plots of the meta-analysis showing the RR in PONV. Figure S5. Forest plots of the meta-analysis showing the RR in hallucination. Figure S6. Forest plots of the meta-analysis showing the RR in itching. Figure S7. Forest plots of the meta-analysis showing the RR in nightmare. Figure S8. Forest plots of the meta-analysis showing the RR in respiratory depression. Figure S9. Forest plots of the meta-analysis showing the RR in dizziness. Figure S10. Forest plots of the meta-analysis showing the RR in requiring analgesia. Reference [39] is cited in the Supplementary Materials.
