# Supplementary material for: Efficacy and Safety of Esketamine in Patients Undergoing Laparoscopic Cholecystectomy: A Systematic Review and Meta-Analysis of Randomized Controlled Trials
Source: J Clin Med. 2026 Jun 24;15(13):4902. doi: 10.3390/jcm15134902 (PMC13361626; doi:10.3390/jcm15134902)
Supplement: Supplementary file 1 [file jcm-15-04902-s001.zip › jcm-4315282-supplementary.pdf]

SUPPLEMENTAL TABLE AND FIGURE LEGENDS

Table S1. PRISMA 2020 checklist

Table S2. Detailed search strategy for each database during the systematic search phase.

Table S3. List of excluded studies during the full-text screening step.

Table S4. Detailed summary of anesthesia and surgical protocols of the included trials.

Figure S1. Forest plots of the meta-analysis showing the MD in wake-up time.

Figure S2. Forest plots of the meta-analysis showing the MD in recovery time.

Figure S3. Forest plots of the meta-analysis showing the MD in sleep quality score.

Figure S4. Forest plots of the meta-analysis showing the RR in PONV.

Figure S5. Forest plots of the meta-analysis showing the RR in hallucination.

Figure S6. Forest plots of the meta-analysis showing the RR in itching.

Figure S7. Forest plots of the meta-analysis showing the RR in nightmare.

Figure S8. Forest plots of the meta-analysis showing the RR in respiratory depression.

Figure S9. Forest plots of the meta-analysis showing the RR in dizziness.

Figure S10. Forest plots of the meta-analysis showing the RR in requiring analgesia.

Table S1. PRISMA 2020 checklist.

Section and Topic	Item #	Checklist item	Location where item is reported
**TITLE**			
Title	1	Identify the report as a systematic review.	1
**ABSTRACT**			
Abstract	2	See the PRISMA 2020 for Abstracts checklist.	NA
**INTRODUCTION**			
Rationale	3	Describe the rationale for the review in the context of existing knowledge.	3
Objectives	4	Provide an explicit statement of the objective(s) or question(s) the review addresses.	3-4
**METHODS**			
Eligibility criteria	5	Specify the inclusion and exclusion criteria for the review and how studies were grouped for the syntheses.	5-6
Information sources	6	Specify all databases, registers, websites, organisations, reference lists and other sources searched or consulted to identify studies. Specify the date when each source was last searched or consulted.	5
Search strategy	7	Present the full search strategies for all databases, registers and websites, including any filters and limits used.	5
Selection process	8	Specify the methods used to decide whether a study met the inclusion criteria of the review, including how many reviewers screened each record and each report retrieved, whether they worked independently, and if applicable, details of automation tools used in the process.	6-7
Data collection process	9	Specify the methods used to collect data from reports, including how many reviewers collected data from each report, whether they worked independently, any processes for obtaining or confirming data from study investigators, and if applicable, details of automation tools used in the process.	6-7
Data items	10a	List and define all outcomes for which data were sought. Specify whether all results that were compatible with each outcome domain in each study were sought (e.g. for all measures, time points, analyses), and if not, the methods used to decide which results to collect.	6-7
	10b	List and define all other variables for which data were sought (e.g. participant and intervention characteristics, funding sources). Describe any assumptions made about any missing or unclear information.	6-7
Study risk of bias assessment	11	Specify the methods used to assess risk of bias in the included studies, including details of the tool(s) used, how many reviewers assessed each study and whether they worked independently, and if applicable, details of automation tools used in the process.	7
Effect measures	12	Specify for each outcome the effect measure(s) (e.g. risk ratio, mean difference) used in the synthesis or presentation of results.	7-8
Synthesis methods	13a	Describe the processes used to decide which studies were eligible for each synthesis (e.g. tabulating the study intervention characteristics and comparing against the planned groups for each synthesis (item #5)).	7-8
	13b	Describe any methods required to prepare the data for presentation or synthesis, such as handling of missing summary statistics, or data conversions.	7-8
	13c	Describe any methods used to tabulate or visually display results of individual studies and syntheses.	7-8
	13d	Describe any methods used to synthesize results and provide a rationale for the choice(s). If meta-analysis was performed, describe the model(s), method(s) to identify the presence and extent of statistical heterogeneity, and software package(s) used.	7-8
	13e	Describe any methods used to explore possible causes of heterogeneity among study results (e.g. subgroup analysis, meta-regression).	7-8
	13f	Describe any sensitivity analyses conducted to assess robustness of the synthesized results.	7-8
Reporting bias assessment	14	Describe any methods used to assess risk of bias due to missing results in a synthesis (arising from reporting biases).	7

Section and Topic	Item #	Checklist item	Location where item is reported
Certainty assessment	15	Describe any methods used to assess certainty (or confidence) in the body of evidence for an outcome.	NA
**RESULTS**			
Study selection	16a	Describe the results of the search and selection process, from the number of records identified in the search to the number of studies included in the review, ideally using a flow diagram.	8
	16b	Cite studies that might appear to meet the inclusion criteria, but which were excluded, and explain why they were excluded.	8
Study characteristics	17	Cite each included study and present its characteristics.	9-10
Risk of bias in studies	18	Present assessments of risk of bias for each included study.	13
Results of individual studies	19	For all outcomes, present, for each study: (a) summary statistics for each group (where appropriate) and (b) an effect estimate and its precision (e.g. confidence/credible interval), ideally using structured tables or plots.	11-13
Results of syntheses	20a	For each synthesis, briefly summarise the characteristics and risk of bias among contributing studies.	11-13
	20b	Present results of all statistical syntheses conducted. If meta-analysis was done, present for each the summary estimate and its precision (e.g. confidence/credible interval) and measures of statistical heterogeneity. If comparing groups, describe the direction of the effect.	11-13
	20c	Present results of all investigations of possible causes of heterogeneity among study results.	11-13
	20d	Present results of all sensitivity analyses conducted to assess the robustness of the synthesized results.	11-13
Reporting biases	21	Present assessments of risk of bias due to missing results (arising from reporting biases) for each synthesis assessed.	13
Certainty of evidence	22	Present assessments of certainty (or confidence) in the body of evidence for each outcome assessed.	NA
**DISCUSSION**			
Discussion	23a	Provide a general interpretation of the results in the context of other evidence.	14
	23b	Discuss any limitations of the evidence included in the review.	17
	23c	Discuss any limitations of the review processes used.	17
	23d	Discuss implications of the results for practice, policy, and future research.	14-18
**OTHER INFORMATION**			
Registration and protocol	24a	Provide registration information for the review, including register name and registration number, or state that the review was not registered.	5
	24b	Indicate where the review protocol can be accessed, or state that a protocol was not prepared.	5
	24c	Describe and explain any amendments to information provided at registration or in the protocol.	NA
Support	25	Describe sources of financial or non-financial support for the review, and the role of the funders or sponsors in the review.	NA
Competing interests	26	Declare any competing interests of review authors.	NA
Availability of data, code and other materials	27	Report which of the following are publicly available and where they can be found: template data collection forms; data extracted from included studies; data used for all analyses; analytic code; any other materials used in the review.	NA

From: Page MJ, McKenzie JE, Bossuyt PM, Boutron I, Hoffmann TC, Mulrow CD, et al. The PRISMA 2020 statement: an updated guideline for reporting systematic reviews. BMJ 2021;372:n71. doi:

10.1136/bmj.n71. This work is licensed under CC BY 4.0. To view a copy of this license, visit https://creativecommons.org/licenses/by/4.0/

Table S2. Detailed search strategy for each database during the systematic search phase.

Database	Search Strategy	Filter	Results
PubMed	("Laparoscopy" or "Laparoscopic" or "Peritoneoscopy") AND ("Cholecystectomy" or "gallbladder removal" or "gallbladder surgery") AND ("Esketamine" or "Kataved" or "Spravato" or "(S)-2-(o-chlorophenyl)-2-(methylamino)cyclohexanone" or "L-Ketamine")	All Fields	13
Scopus	("Laparoscopy" or "Laparoscopic" or "Peritoneoscopy") AND ("Cholecystectomy" or "gallbladder removal" or "gallbladder surgery") AND ("Esketamine" or "Kataved" or "Spravato" or "(S)-2-(o-chlorophenyl)-2-(methylamino)cyclohexanone" or "L-Ketamine")	Article title, Abstract, Keywords	24
Web of Science	("Laparoscopy" or "Laparoscopic" or "Peritoneoscopy") AND ("Cholecystectomy" or "gallbladder removal" or "gallbladder surgery") AND ("Esketamine" or "Kataved" or "Spravato" or "(S)-2-(o-chlorophenyl)-2-(methylamino)cyclohexanone" or "L-Ketamine")	All Fields	36
Embase	("Laparoscopy" or "Laparoscopic" or "Peritoneoscopy") AND ("Cholecystectomy" or "gallbladder removal" or "gallbladder surgery") AND ("Esketamine" or "Kataved" or "Spravato" or "(S)-2-(o-chlorophenyl)-2-(methylamino)cyclohexanone" or "L-Ketamine")	Article title, Abstract, Keywords	47
CENTRAL	("Laparoscopy" or "Laparoscopic" or "Peritoneoscopy") AND ("Cholecystectomy" or "gallbladder removal" or "gallbladder surgery") AND ("Esketamine" or "Kataved" or "Spravato" or "(S)-2-(o-chlorophenyl)-2-(methylamino)cyclohexanone" or "L-Ketamine")	Article title, Abstract, Keywords	39

Table S3. List of excluded studies during the full-text screening step.

Study ID	Title	Reason of exclusion
Deng et al. (2024)	Comparison of the analgesic effect of dezocine and esketamine in combination with sufentanil respectively after laparoscopic cholecystectomy: a prospective randomized controlled study	Active comparator-Dezocine
Yu et al. (2023)	Opioid-Free Anesthesia for Pain Relief After Laparoscopic Cholecystectomy: A Prospective Randomized Controlled Trial	Active comparator-Remifentanil
Hu et al. (2024)	The Impact of Esketamine Combined with Dexmedetomidine on Laparoscopic Gallbladder Surgery: A Randomized Controlled Trial	Esketamine combined with Dexmedetomidine

Table S4. Detailed summary of anesthesia and surgical protocols of the included trials.

Study ID	Premedications	Anesthesia protocol	Postmedications	Surgical protocol
Li et al. 2023	No specific oral premedication reported	Induction: midazolam 0.05 mg/kg, sufentanil 0.4 µg/kg, propofol 0.8–1.2 mg/kg, cisatracurium 0.2 mg/kg. Research drug 1 (saline / esketamine 0.2 or 0.3 mg/kg) IV bolus 1 min before intubation. Mechanical ventilation: O_2 flow 1.5–2 L/min, VT 6–8 mL/kg, $PETCO_2$ 35–45 mmHg. Maintenance (pure IV): propofol 2–6 mg/kg/h and remifentanil 0.1–0.3 µg/kg/min titrated to BIS 40–60. Research drug 2 (esketamine 0.2 or 0.3 mg/kg/h or saline) infused until gallbladder removal	During the surgery, hemodynamics controlled with norepinephrine 20–50 µg or atropine 0.2–0.5 mg as needed. At end of the surgery: Flurbiprofen axetil 50 mg IV was given. Additional flurbiprofen 50 mg if NRS >3. Tropisetron 5 mg if nausea/vomiting. Extubation after recovery of consciousness and muscle strength. Transfer to the ward was considered when Aldrete >9.	All patients underwent elective laparoscopic cholecystectomy
Wu et al. 2025	Before induction: Tropisetron 5 mg IV + Dexamethasone 10 mg IV for PONV prophylaxis	All patients received TIVA. Induction: propofol 1–2 mg/kg, sufentanil 2–4 µg/kg, cisatracurium 0.2 mg/kg. Maintenance: propofol TCI 4–6 mg/kg/h + remifentanil 0.2–0.6 µg/kg/h titrated to keep BP/HR within ±20% baseline. Esketamine group: continuous infusion 0.5 mg/kg/h, stopped 30 min before end of surgery. Placebo: equal saline. Local analgesia: surgeon infiltrated 20 mL of 0.375% ropivacaine at incision at start of surgery. Ventilation: VT 6–8 mL/kg.	If VAS >4, Parecoxib 40 mg IV was given. If it was inadequate, Dezocine 5 mg IV was given	All patients underwent elective laparoscopic cholecystectomy
Xu et al. 2023	No specific oral premedication reported	General anesthesia induced with midazolam 0.03–0.04 mg/kg, etomidate 0.2–0.3 mg/kg, sufentanil 0.3–0.5 µg/kg, and cisatracurium 0.15–0.2 mg/kg for intubation. Before skin incision, Group E received variable doses of IV esketamine, Group C received saline. Maintenance with propofol 4–6 mg/kg/h, sevoflurane 1–1.5%, remifentanil 0.2–0.3 µg/kg/min, and cisatracurium 0.1–0.15 mg/kg/h. Sevoflurane and cisatracurium stopped during skin closure	Parecoxib 40 mg IV when pneumoperitoneum stopped. 0.5% ropivacaine local infiltration at incision at closure. HR managed with atropine and esmolol. BP managed with dopamine and urapidil as needed. Residual block reversed with neostigmine 0.02 mg/kg and atropine 0.01 mg/kg if the TOF ratios were 1.0. Rescue analgesia in PACU: morphine 0.1 mg/kg if VAS >4.	All patients underwent elective laparoscopic cholecystectomy

Zhang et al. 2025	No specific oral premedication reported	Induction: sufentanil 0.4 µg/kg, etomidate 0.2 mg/kg, rocuronium 0.9 mg/kg for intubation. After intubation, study drug administered: esketamine 0.3 mg/kg IV or saline. Maintenance: propofol 60–120 µg/kg/min, remifentanil 0.2–0.5 µg/kg/min, and sevoflurane 1–2%. BIS maintained 40–60. Ventilation to keep $PETCO_2$ 35–45 mmHg	BP controlled with phenylephrine or nitroglycerin. Bradycardia (<45 bpm) treated with atropine 0.25 mg. After surgery: Flurbiprofen axetil 50 mg IV. Bilateral transversus abdominis plane block under ultrasound with 20 mL of 0.375% ropivacaine each side. Rescue analgesia if VAS >3: sufentanil 2–3 µg/kg	All patients underwent elective laparoscopic cholecystectomy
Zhang et al. 2022	No specific oral premedication reported	All patients received TIVA. During induction: esketamine 0.2 mg/kg IV (or saline) before propofol. Induction with midazolam 0.04 mg/kg, atropine 0.01 mg/kg, propofol 1.5 mg/kg, sufentanil 0.5 µg/kg, rocuronium 0.6 mg/kg. Maintenance: propofol infusion 6 mg/kg/h and remifentanil 0.1–0.3 µg/kg/min adjusted to vitals. No volatile agent used	At end of the surgery: Flurbiprofen axetil 50 mg IV was given. If NRS >4, sufentanil 0.05 µg/kg was given	All patients underwent elective laparoscopic cholecystectomy
Zhao et al. 2024	No specific oral premedication reported	Induction (IV): penehyclidine 0.5 mg, dexamethasone 10 mg, midazolam 0.03–0.06 mg/kg, sufentanil 0.4 µg/kg, etomidate 0.2–0.3 mg/kg, cis-atracurium 0.1–0.3 mg/kg. Maintenance (IV): propofol 0.1–0.2 mg/kg·min and remifentanil 0.1–0.2 µg/kg·min, adjusted to BIS. Cis-atracurium 0.02–0.03 mg/kg q30 min. Ventilation: RR 12–14/min, $PETCO_2$ 35–45 mmHg, O_2 flow 2 L/min. Study drug (pre-incision): NS: saline 0.5 mg/kg and infusion 2 µg/kg·min; LS: esketamine 0.5 mg/kg and 2 µg/kg·min; HS: esketamine 0.5 mg/kg and 4 µg/kg·min.	If NRS ≥4, sufentanil 0.05 µg/kg IV was given; total within three hours not to exceed 0.1 µg/kg.	All patients underwent elective laparoscopic cholecystectomy

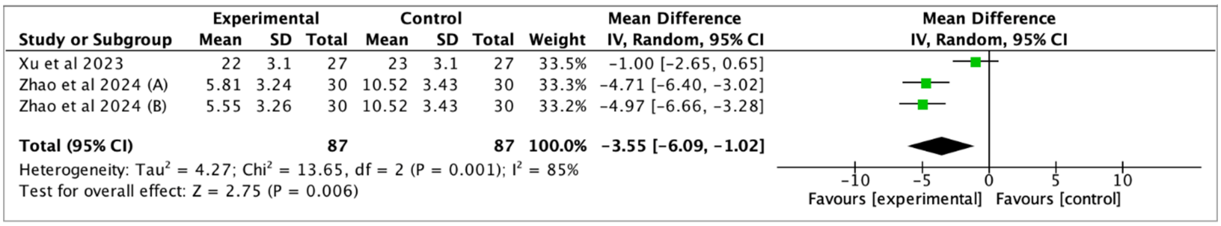

Figure S1. Forest plots of the meta-analysis showing the MD in wake-up time.

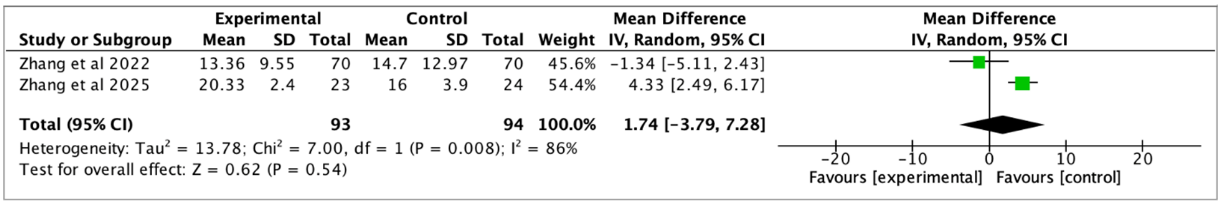

Figure S2. Forest plots of the meta-analysis showing the MD in recovery time.

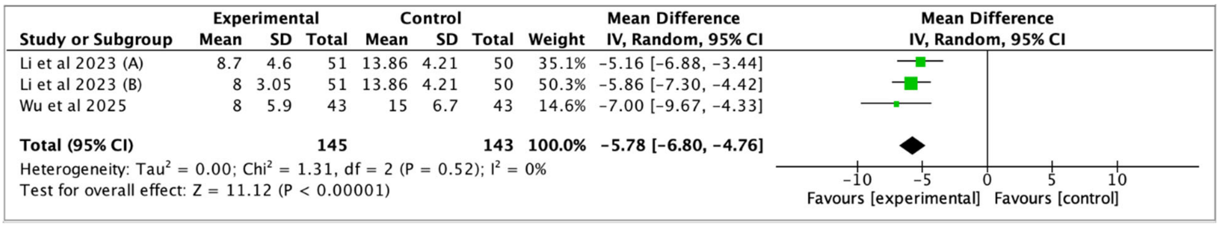

Figure S3. Forest plots of the meta-analysis showing the MD in sleep quality score.

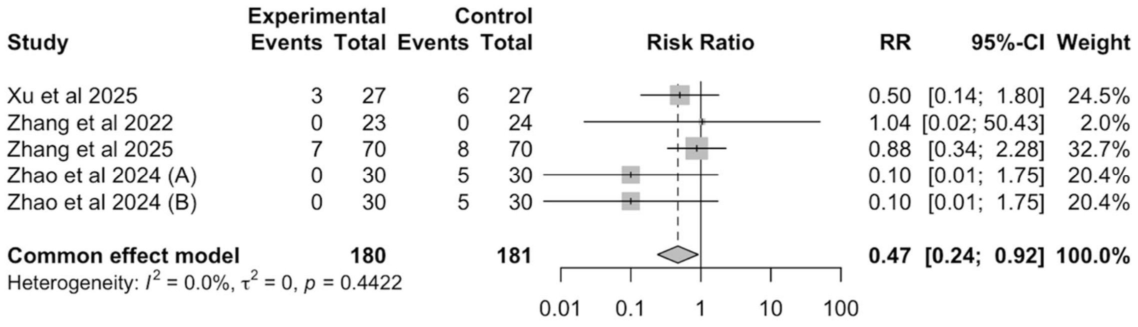

Figure S4. Forest plots of the meta-analysis showing the RR in PONV.

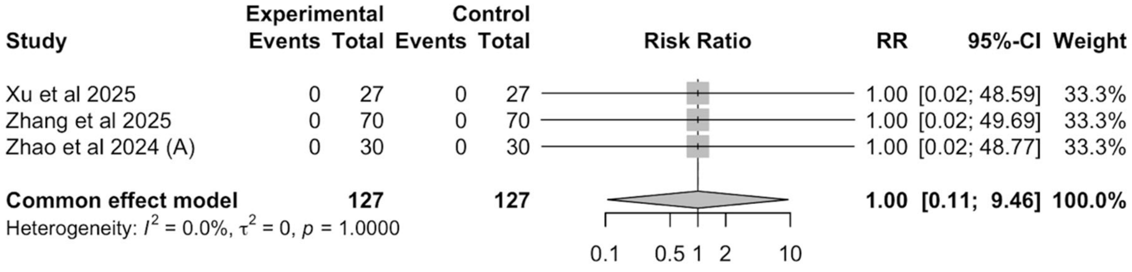

Figure S5. Forest plots of the meta-analysis showing the RR in hallucination.

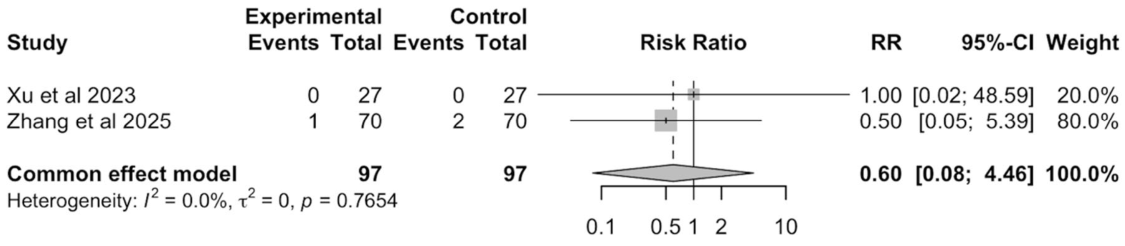

Figure S6. Forest plots of the meta-analysis showing the RR in itching.

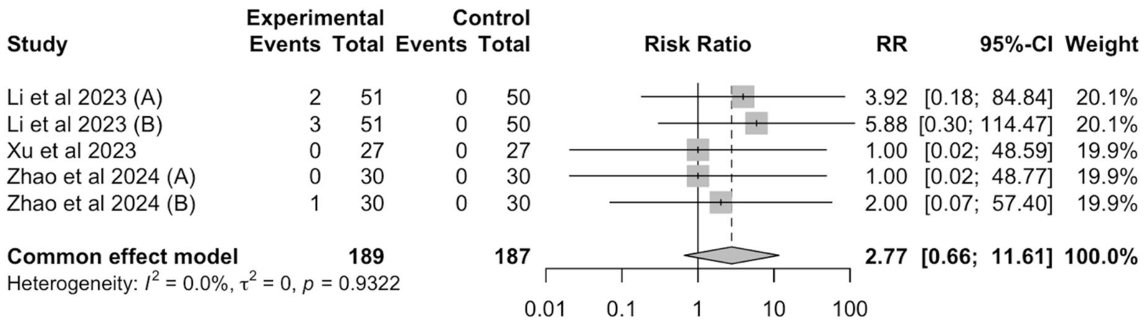

Figure S7. Forest plots of the meta-analysis showing the RR in nightmare.

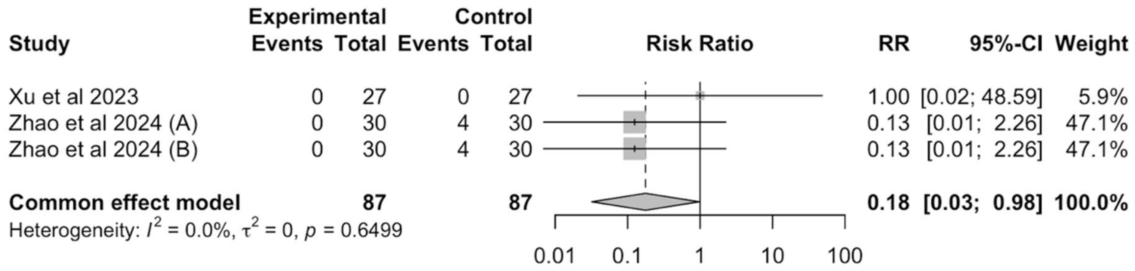

Figure S8. Forest plots of the meta-analysis showing the RR in respiratory depression.

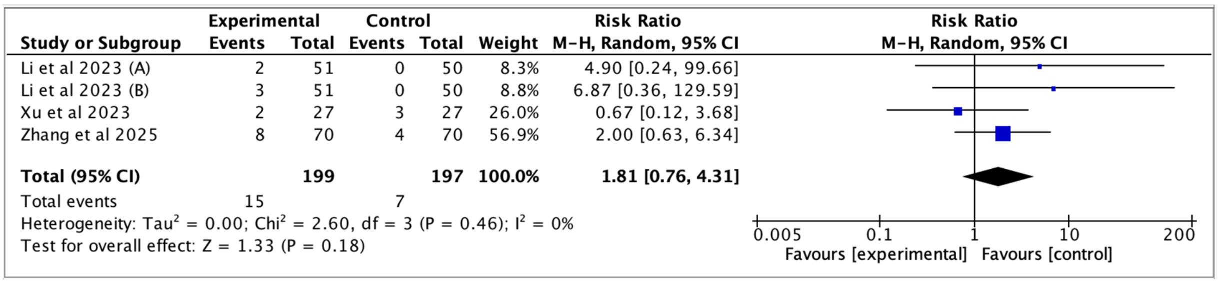

Figure S9. Forest plots of the meta-analysis showing the RR in dizziness.

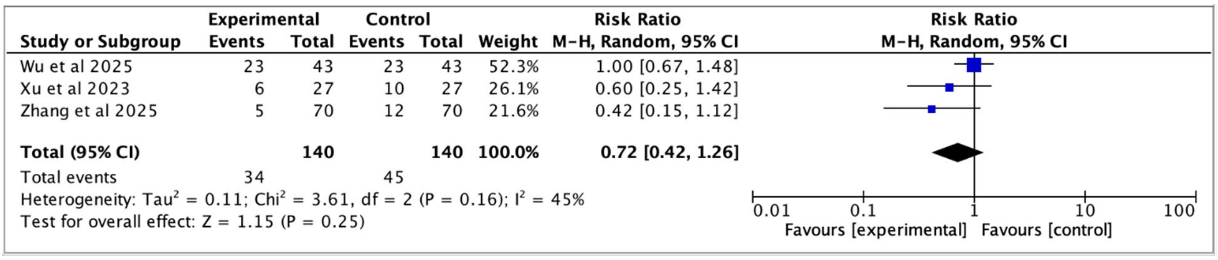

Figure S10. Forest plots of the meta-analysis showing the RR in requiring analgesia.
